# Risk factors for seizures in the vigorous term neonate: A population-based register study of singleton births in Sweden

**DOI:** 10.1371/journal.pone.0264117

**Published:** 2022-02-17

**Authors:** Malin Dickmark, Johan Ågren, Lena Hellström-Westas, Maria Jonsson

**Affiliations:** 1 Department of Obstetrics and Gynecology, Uppsala University Hospital, Uppsala, Sweden; 2 Department of Women’s and Children’s Health, Uppsala University, Uppsala, Sweden; University of Oslo, NORWAY

## Abstract

**Background:**

Neonatal seizures have been associated with increased mortality and impaired neurodevelopment and, knowledge about risk factors may be useful for prevention. Clear associations have been established between labor-related risk factors and seizures in asphyxiated neonates. However, there is limited information about why some vigorous term-born infants experience seizures.

**Objectives:**

Our aim was to assess antepartum and intrapartum risk factors for seizures in vigorous term-born neonates.

**Methods:**

This was a national cohort study of singleton infants born at term in Sweden from 2009–2015. Vigorous was defined as an Apgar score of at least 7 at 5 and 10 minutes. Data on the mothers and infants were obtained from the Swedish Medical Birth Register and the Swedish Neonatal Quality Register. A diagnosis of neonatal seizures was the main outcome measure and the exposures were pregnancy and labor variables. Logistic regression analysis was used and the results are expressed as adjusted odds ratios (aOR) with 95% confidence intervals (CI).

**Results:**

The incidence of neonatal seizures was 0.81/1,000 for 656 088 births. Seizures were strongly associated with obstetric emergencies (aOR 4.0, 95% CI 2.2–7.4), intrapartum fever and/or chorioamnionitis (aOR 3.4, 95% CI 2.1–5.3), and intrapartum fetal distress (aOR 3.0, 95% CI 2.4–3.7). Other associated intrapartum factors were: labor dystocia, occiput posterior position, operative vaginal delivery, and Cesarean delivery. Some maternal factors more than doubled the risk: a body mass of more than 40 (aOR 2.6, 95% CI 1.4–4.8), hypertensive disorders (aOR 2.3, 95% CI 1.7–3.1) and diabetes mellitus (aOR 2.6, 95% CI 1.7–4.1).

**Conclusion:**

A number of intrapartum factors were associated with an increased risk of seizures in vigorous term-born neonates. Obstetric emergencies, intrapartum fever and/or chorioamnionitis and fetal distress were the strongest associated risks. The presence of such factors, despite a reassuring Apgar score could prompt close surveillance.

## Introduction

The risk for seizures in term-born infants born after low-risk pregnancies is low, at around 0.2 per 1,000 deliveries [1]. However, neonatal seizures affect around 1.3 per 1,000 live-born infants [2–5] and the majority of these seizures occur during the first few days of life [6]. Most of these seizures appear to be secondary to perinatal and neonatal insults, and they have been associated with increased mortality and the risk of impaired neurodevelopment and epilepsy in survivors [4, 7].

Birth asphyxia is the most common etiology of neonatal seizures in term infants, and it has been associated with maternal and obstetrical complications before and during delivery [6–9]. Obstetric risk factors for neonatal seizures include nulliparity, diabetes mellitus, obesity, smoking, post-term deliveries, fever or infections in labor, asphyxia, a prolonged second stage, operative vaginal delivery, and shoulder dystocia [1, 3, 5, 6, 10–12]. Although the causes of seizures are well defined in a significant proportion of infants, little is known about the factors that can predict seizures in term infants that are vigorous at birth [7, 8]. A case-control study that assessed the risk factors in infants who had seizures, but did not have hypoxic ischemic encephalopathy, indicated that there may have been modifiable obstetric risks associated with the seizures [13].

We hypothesized that neonatal seizures in vigorous term-born infants, could be associated with less well-defined obstetric events, than in other seemingly vulnerable infants, including maternal risk factors and intrapartum events. That is why the aim of this population-based study was to investigate potentially recognisable maternal and obstetrical risks in term infants who were vigorous at birth, but went on to have neonatal seizures.

## Materials and methods

This was a nation-wide Swedish cohort study which was based on an anonymized dataset of 710,494 singleton infants who were born alive and at term, without congenital malformations, from 2009 to 2015. The study was approved by the regional ethics review board at Uppsala University, Uppsala, Sweden (No 2015/156). The data were retrieved from two national quality registers, the Swedish Medical Birth Register (MBR) and the Swedish Neonatal Quality Register (SNQ).

**Fig 1:**
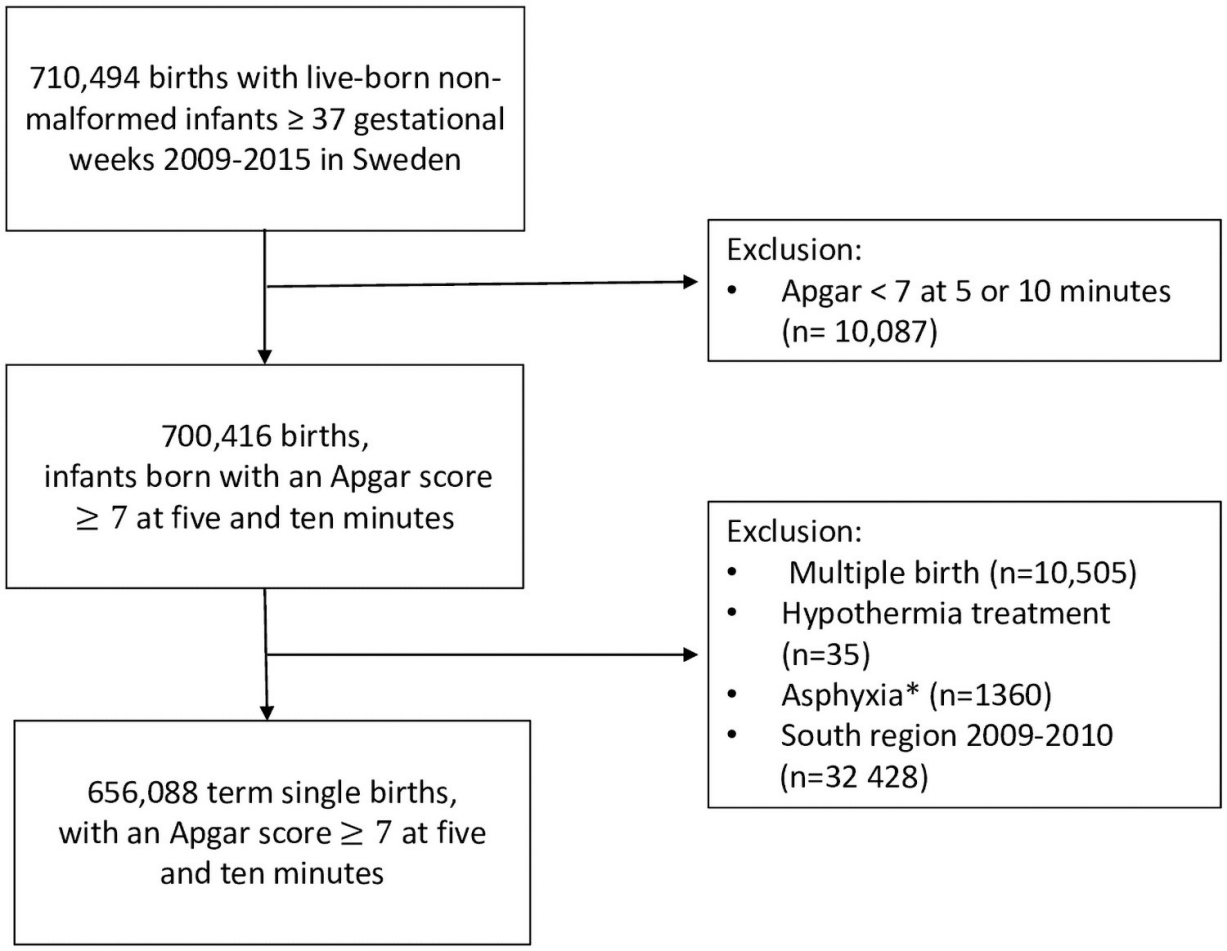
Study population. * P21 (ICD 10).

We defined vigorous infants as those who had an Apgar score of at least 7 at 5 and 10 minutes [14] and who did not have a diagnosis of asphyxia. The study flowchart (Fig 1) shows that we excluded 10 087 infants with low Apgar scores of less than 7 at 5 and 10 minutes. We also excluded 10 505 multiple pregnancies, 35 infants who received hypothermia treatment and 1360 diagnosed with birth asphyxia according to the International Classification of Diseases, Tenth Revision (ICD-10) code P21. The healthcare region that covers southern Sweden did not start entering data into the SNQ until 2011 and 32 428 births from that region were therefore excluded from the data for 2009–2010. This meant that the final study population comprised 656 088 liveborn term singleton infants with a reassuring Apgar score at 7 at 5 and 10 minutes and no diagnosis of asphyxia.

The MBR, which was established in 1973, contains information on antenatal, obstetrical, and neonatal care and diagnoses for more than 98% of births in Sweden. It receives prospectively collected data from all healthcare providers in the country who complete standardised forms. The coverage and validity of most of the variables are high [15]. Maternal care is provided free of charge to all women in Sweden which contributes to high internal validity. The SNQ is also a national register which was established in 2001 and holds information on all newborn infants that are admitted to Swedish neonatal care units [16]. The data are prospectively collected using standardized forms. The SNQ has demonstrated excellent agreement with other Swedish registers [17]. Data from the MBR and the SNQ were linked by the Swedish National Board of Health and Welfare performed for this study, using the unique personal identity numbers assigned to all Swedish citizens.

### Exposure variables

Pre-defined exposure variables were retrieved from the MBR and these included maternal characteristics, data on the birth, and basic information on the newborn infants. Maternal ethnicity was categorized as Nordic or non-Nordic. Co-habitation, smoking, and infertility treatment were dichotomized into yes or no. Parity was self-reported as nulliparous or parous, with or without previous Cesarean sections. Maternal age was divided into: <20 years, 21–34 years, and >35 years. Self-reported maternal height was divided into: ≤ 155 cm, 156–160 cm, 161–172 cm, and >172 cm. The mother’s early pregnancy body mass index (BMI) defined as her weight in kg/height in m^2^ was calculated based on data measured at the first antenatal visit. It was then categorized into the groups defined by the World Health Organization [18]: underweight (<18.5 kg/m^2^), normal weight (18.5–24.9 kg/m^2^), pre-obesity (25–29.9 kg/m^2^), obesity class 1 (30–34.9 kg/m^2^), obesity class 2 (35–39.9 kg/m^2^), and obesity class 3 (>40 kg/m^2^).

The ICD-10 codes retrieved for hypertension were chronic hypertension (O10), gestational hypertension (O13.9), preeclampsia (O14), or eclampsia (O15). Hypertensive disorders were regarded as both a compound variable and a differentiated variable. Diabetes mellitus (O24) was defined as either pregestational or gestational diabetes mellitus. We also retrieved the ICD-10 codes for other relevant maternal conditions or pregnancy complications. These ICD-10 codes included thyroid disease (O99.2; E22–E23), epilepsy (O99.3; G40), asthma (O99.5; J45), intrahepatic cholestasis (O26.6), and isoimmunization (O36.0; O36.1). All diseases were dichotomous variables.

Labor characteristics were also collected from the MBR. Gestational age was categorized as 37–40 weeks, 41–42 weeks, or >42 weeks. A number of ICD-10 codes that relate to induced labor and medical complications were treated as dichotomous variables. These included premature rupture of membranes (O75.6), labor dystocia (O62, O63), meconium-stained amniotic fluid (O68.1), intrapartum fever (O75.2) and/or chorioamnionitis (O41.1), fetal distress in labor and delivery (O68), and umbilical cord complications (O69). Fetal distress was when the labor or delivery was complicated by a fetal heart rate anomaly, biochemical evidence of fetal distress (scalp blood sampling), and/or presence of meconium in the amniotic fluid. The fetal position at birth was coded as occiput anterior, occiput posterior, breech, or other. The mode of delivery was spontaneous vaginal delivery, operative vaginal delivery using a vacuum or forceps or a prelabor, or emergency Cesarean section. Obstetric emergencies included umbilical cord prolapse (O69.0), uterine rupture (O71.1), placental abruption (O45), eclampsia (O15), and/or shoulder dystocia (O66.0). The infant characteristics included the baby’s sex, whether they had a head circumference <37 cm or >37 cm, whether their birthweight was <3000 g, 3000–4000 g, or >4000 g, and whether they were classified as small for gestational age (SGA), large for gestational age (LGA), or appropriate for gestational age (AGA). SGA was defined as a birthweight that was <2 standard deviations (SD) for gestational age according to the Swedish fetal growth chart and LGA was >2 SD [19].

### Outcome

The investigated outcome was whether the attending physician had submitted a diagnosis of neonatal seizures indicated by the ICD-10 code P90 to the MBR or SNQ. Swedish neonatal intensive care units normally use an amplitude-integrated EEG (aEEG) monitoring or conventional electroencephalogram (EEG) to diagnose neonatal seizures as recommended by national guidelines for neonatal seizure management [20]. aEEG monitoring is frequently used when there are clinically suspected seizures or other signs of encephalopathy. The SNQ includes a box that can be ticked when a conventional EEG took place.

### Statistics

The numbers of observations, in numbers and percentages, and rates per 1,000 are presented with descriptive statistics. We assessed the risk factors for neonatal seizures using bivariate logistic regression and these are expressed as odds ratios (OR) with 95% confidence intervals (95% CI). When multivariable analyses were carried out, the estimates were adjusted for parity, maternal height, BMI, gestational age, and infant birth year and the results are presented as adjusted ORs (aORs). A sensitivity analysis was performed by excluding SGA and LGA births because these have increased risks for complications during pregnancy and labor [21–23]. A two-sided p-value of less than 0.05 was considered statistically significant. The results were analysed using IBM^©^ SPSS^©^ Statistics, version 26.

## Results

The data for 656 088 term singleton infants were analysed and this showed that 534 vigorous neonates had seizures within the first 28 days of life. This corresponded to an incidence of 0.81 per 1,000 live-born neonates. Just under two-thirds (62%) of these seizures were verified by conventional EEG. Table 1 compares the maternal demographic and antepartum characteristics of the neonates who did and did not have seizures. This shows that there were no significant differences in the mother’s ethnicity, age, co-habitation, or smoking. Nulliparity was more common in infants with seizures than those without seizures (58.2% versus 43.3%) and so were maternal height ≤ 161 cm, obesity classes 2 or 3, or a history of infertility. Seizures were more common in cases of maternal hypertensive disorders and, these included preeclampsia, but not chronic hypertension. Maternal epilepsy, or diabetes mellitus were also more common in the group with seizures. However, maternal thyroid disease, asthma, intrahepatic cholestasis, and isoimmunization were not associated with neonatal seizures (data not shown).

**Table 1:** Maternal demographic and antepartum characteristics.

	Study population	Infants with seizures	Infants with no seizures
	n = 656 088	n = 534 (%)	n = 655 554 (%)
Parity			
Nulliparity	284,041	311 (58.2)	283,730 (43.3)
Parous without previous CS	288,406	143 (26.8)	288,263 (43.9)
Parous with previous CS	66,073	69 (12.9)	66,004 (10.1)
Missing	17,568	11 (2.1)	17,557 (2.7)
Maternal age (years)			
≤20	16,772	13 (2.4)	16,759 (2.5)
21–34	495,556	405 (75.8)	495,151 (75.5)
≥35	143,758	116 (21.7)	143,642 (21.9)
Missing	2	0	2
Maternal height (cm)			
≤155	21,897	28 (5.2)	21,869 (3.3)
156–160	103,872	106 (19.9)	103,776 (15.8)
161–172	399,749	301 (56.4)	399,448 (60.9)
>172	107,044	70 (13.1)	106,974 (16.3)
Missing	23,526	29 (5.4)	23,497 (3.6)
Early pregnancy BMI (kg/m^2^)			
<18.5	15,558	6 (1.1)	15,552 (2.4)
18.5–24.9	364,108	262 (49.1)	363,846 (55.5)
25–29.9	160,474	140 (26,2)	160,334 (24.5)
30–34.9	55,008	57 (10.7)	54,951 (8.4)
35–39.9	17,066	22 (4.1)	17,044 (2.6)
≥40	5,974	11 (2.1)	5,963 (0.9)
Missing	37,900	36 (6.7)	37,864 (5.7)
Co-habiting	590,125	472 (88.4)	589,653 (89.9)
Missing	25,461	26 (4.9)	25,435 (3.9)
Smoking	36,357	35 (6.5)	36,322 (5.5)
Missing	21,656	25 (4.7)	21,631 (3.3)
Epilepsy	3,263	6 (1.1)	3,257 (0.5)
Infertility treatment	19,599	26 (4.9)	19,573 (2.9)
Hypertensive disorders^a^	25,389	54 (10.1)	25,335 (3.7)
Chronic hypertension	2,332	2 (0.4)	2,330 (0.4)
Gestational hypertension	8,240	15 (2.8)	8,225 (1.3)
Preeclampsia/eclampsia	15,193	37 (6.9)	15,156 (2.3)
Diabetes mellitus^b^	10,021	23 (4.3)	9,998 (1.5)

^a^Chronic hypertension, gestational hypertension, preeclampsia or eclampsia.

^b^Pregestational or gestational diabetes mellitus.

BMI: body mass index; CS: Cesarean section.

The adjusted analyses in Table 2 show that nulliparity more than doubled the risk for neonatal seizures (aOR 2.2, 95% CI 1.8–2.7) and a maternal height of ≤155 cm increased the risk by 60% (aOR 1.6, 95% CI 1.1–1.6). Maternal epilepsy, hypertensive disorders, and diabetes mellitus were all risk factors for neonatal seizures. The highest risks overall were found in women with diabetes mellitus (aOR 2.6, 95% CI, 1.7–4.1) or BMI class 3 (aOR 2.6, 95% CI 1.4–4.8). The sensitivity analysis, which only included infants born AGA (S1 Table), showed that the aORs for maternal characteristics were largely unchanged and hypertensive disorders remained a risk factor (aOR 2.0, 95% CI 1.4–2.8). However, epilepsy and diabetes mellitus were not associated with neonatal seizures.

**Table 2:** Frequencies, crude and adjusted odds ratios for neonatal seizures by demographic and antepartum characteristics.

Factors	Total number n = 656,088	Seizures n = 534	Rate /1,000	Crude OR (95% CI)	Adjusted OR^1^ (95% CI)
Parity					
Nulliparity	284,041	311	1.1	2.2 (1.8, 2.7)	2.2 (1.8, 2.7)
Parous without previous CS	288,406	143	0.5	1.0	1.0
Parous with previous CS	66,073	69	1.0	2.1 (1.6, 2.8)	1.9 (1.5, 2.7)
Maternal height (cm)					
≤155	21,897	28	1.3	1.7 (1.2, 2.5)	1.6 (1.1, 2.5)
156–160	103,872	106	1.0	1.4 (1.1, 1.7)	1.3 (1.0, 1.6)
161–172	399,749	301	0.8	1.0	1.0
>172	107,044	70	0.7	0.9 (0.7, 1.1)	0.9 (0.7, 1.2)
Early pregnancy BMI (kg/m^2^)					
<18.5	16,275	6	0.4	0.5 (0.2, 1.2)	0.5 (0.2, 1.2)
18.5–24.9	382,058	262	0.7	1.0	1.0
25–29.9	168,711	140	0.8	1.2 (0.9, 1.5)	1.2 (0.9, 1.5)
30–34.9	57,704	57	0.9	1.4 (1.1, 1.9)	1.4 (1.0, 1.9)
35–39.9	17,927	22	1.2	1.8 (1.2, 2.8)	1.9 (1.2, 2.8)
≥40	6,294	11	1.7	2.6 (1.4, 47)	2.6 (1.4, 4.8)
Infertility treatment	19,599	26	1.3	1.7 (1.1, 2.5)	1.5 (1.0, 2.3)
Epilepsy	3,263	6	1.8	2.3 (1.0, 5.1)	2.3 (1.0, 5.1)
Hypertensive disorders^a^	25,389	54	2.1	2.8 (2.1, 3.7)	2.3 (1.7, 3.1)
Diabetes mellitus^b^	10,021	23	2.3	2.9 (1.9, 4.4)	2.6 (1.7, 4.1)

^a^Chronic hypertension, gestational hypertension, preeclampsia, or eclampsia.

^b^Pregestational or gestational diabetes mellitus.

^1^Adjusted for parity, maternal height, BMI, gestational age, and infant birth year.

BMI: body mass index; CI: confidence interval; CS: Cesarean section; OR: odds ratio.

The intrapartum risk factors for neonatal seizures are displayed in Table 3. Fetal distress, fever and/or chorioamnionitis, and obstetric emergencies increased the risk. The highest risk estimates were for shoulder dystocia (OR 6.8, 95% CI 3.2–14.3) and placental abruption (OR 5.3, 95% CI 1.9–14.1). Other significant risks for neonatal seizures included induced labor, gestational age ≥42 weeks, labor dystocia, meconium in the amniotic fluid, fetal malposition, head circumference >37 cm, and being LGA or SGA. Operative vaginal deliveries and emergency Cesarean sections increased the risk by almost three times. Prelabor Cesarean sections were also associated with seizures.

**Table 3:** Frequencies, crude and adjusted odds ratios for neonatal seizures by intrapartum factors.

	Seizures N = 534	Rate/1000	No seizure N = 655 554	P value	Crude OR (95% CI)	Adjusted OR^1^ (95% CI)
Induced labor	119 (22.3)	1.3	93 934 (14.3)	< 0.001	1.7 (1.4–2.1)	1.6 (1.3–2.1)
Gestational age, weeks				0.04		
37–40	380 (71.2)	0.7	482 749 (73.6)		1.0	1.0
≥ 41	101 (18.9)	0.8	125 961(19.2)		1.0 (0.8–1.3)	0.9 (0.7–1.2)
≥ 42	53 (9.9)	1.1	46 844 (7.1)		1.4 (1.1–1.9)	1.2 (0.9–1.6)
Premature rupture of membranes	48 (9.0)	0.9	48 682 (7.4)	0.17	1.2 (0.9–1.6)	1.1 (0.8–1.5)
Dystocia	102 (19.1)	1.3	75 763 (11.6)	<0.001	1.8 (1.5–2.2)	1.4 (1.1–1.8)
Meconium	16 (3.0)	2.7	5 825 (0.9)	<0.001	3.4 (2.1–5.7)	3.4 (2.0–5.5)
Intrapartum fever and/or chorioamnionitis	20 (3.7)	3.4	5 848 (0.9)	<0.001	4.3 (2.7–6.7)	3.4 (2.1–5.3)
Position at birth				<0.001		
Occiput anterior	453 (85)	0.8	585 988 (89.4)		1.0	1.0
Occiput posterior	38 (7.1)	1.3	28 902 (4.4)		1.7 (1.2–2.4)	1.5 (1.1–2.1)
Breech lie	14 (2.6)	0.8	15 853 (2.4)		1.1 (0.6–1.9)	1.1 (0.6–1.8)
Other	19 (3.5)	1.6	11 439 (1.7)		2.1 (1.3–3.4)	1.6 (1.0–2.7)
Missing	10 (1.9)		13 372 (2.0)		-	-
Mode of delivery				<0.001		
Spontaneous vaginal	303(56.7)	0.6	502 733 (76.6)		1.0	1.0
Vacuum or forceps	80 (14.9)	1.7	46 107 (7.0)		2.9 (2.2–3.7)	2.2 (1.7–2.9)
Emergency CS	80(14.9)	1.8	44 161 (6.7)		3.0 (2.3–3.8)	2.4 (1.8–3.1)
Prelabor CS	58 (10.8)	1.1	51 695 (7.8)		1.8 (1.4–2.4)	1.5 (1.1–2.1)
Missing	13 (2.4)		10 858 (1.6)			
Threatening asphyxia	110 (20.6)	2.4	45 042 (6.9)	<0.001	3.5 (2.8–4.3)	3.0 (2.4–3.7)
Obstetric emergency	13 (2.4)	3.8	3 399 (0.5)	<0.001	4.8 (2.8–8.3)	4.0 (2.2–7.4)
Shoulder dystocia	7 (1.3)	5.4	1 279 (0.2)	<0.001	6.8 (3.2–14.3)	6.4 (2.8–14.3)
Placental abruption	4 (0.7)	4.2	938 (0.1)	<0.001	5.3 (1.9–14.1)	3.8 (1.2–11.9)
Cord complication	4 (0.7)	1.7	2 264 (0.4)	0.11	2.2 (0.8–5.8)	2.2 (0.8–5.8)
Birth weight, g				0.39		
< 3000g	59 (11.0)	0.7	81 398 (12.4)		0.8 (0.6–1.1)	0.9 (0.6–1.2)
3000-4000g	382 (71.5)	0.8	451 465 (68.9)		1.0	1.0
>4000g	93 (17.4)	0.7	122 691(18.7)		0.9 (0.7–1.1)	0.9 (0.7–1.2)
Head circumference						
>37cm	114 (22.5)	1.1	101 705 (15.6)		1.6 (1.3–1.9)	1.5 (1.2–1.8)
LGA^a^	33 (6.2)	1.5	22 226 (3.4)	<0.001	1.8 (1.3–2.7)	1.9 (1.3–2.8)
SGA^b^	23 (4.3)	1.9	11 761 (1.8)	<0.001	2.5 (1.6–3.7)	1.8 (1.2–2.9)
Male sex	296 (55.4)	0.8	334 234 (51.0)	0.04	1.2 (1.0–1.4)	1.2 (0.9–1.4)

Values are presented as n (%), unless otherwise indicated.

^a^Large for gestational age.

^b^Small for gestational age.

CI: confidence interval; CS: cesarean section; OR: odds ratio.

Most of the risk estimates remained significant in the adjusted analysis (Table 3), except for a gestational age of ≥ 42 weeks and male sex. The highest risk estimates remained with shoulder dystocia (aOR 6.4, 95% CI 2.8–14.3) and placental abruption (aOR 3.8, 95% CI 1.2–11.9). These were even higher for infants born AGA. The adjusted risk for meconium-stained amniotic fluid, intrapartum fever and/or chorioamnionitis, obstetric emergency, and fetal distress were three-fold or higher. The sensitivity analysis for infants born AGA (S2 Table) showed even stronger association between seizures and shoulder dystocia (aOR 7.7, 95% CI 3.2–18.6) and placental abruption (aOR 4.5, 95% CI 1.4–14.1).

## Discussion

This large population-based study found that intrapartum factors posed the highest associated risk for seizures in vigorous neonates. The risks were particularly high when obstetric emergencies, maternal fever or chorioamnionitis were involved. Our results indicated that the intrapartum period was highly relevant when it came to the risk of neonatal seizures in infants born with reassuring Apgar scores. These findings may have important clinical implications, because some of these events could be avoided.

However, it may not be possible to influence risk factors such as BMI class 1 or higher, hypertensive disorders, and diabetes mellitus in the short term. Prevention or improved treatment of these conditions during pregnancy and in labor could potentially reduce the risk of neonatal seizures.

### Strengths and limitations

The population-based design and the large sample size based on national registers were major strengths of this study and this meant that the risks for selection and recall bias were minimized. The size of the cohort enabled us to study a rare outcome and adjust the data for a number of covariates. The MBR is a validated register and contains approximately 98% of all births in Sweden [15]. Maternal care is provided free of charge to all women in Sweden, which contributes to high internal validity of the MBR. The SNQ register covers all neonatal units and data are collected prospectively using standardized forms. It has recently been validated and it demonstrated excellent agreement with other Swedish registers [17].

A number of limitations also need to be considered. For example, a more detailed investigation into the neonatal diagnoses associated with seizures would probably have contributed to increased our understanding of the etiologies. However, this information was not available in our dataset. One limitation was that the registers do not require diagnoses of neonatal seizures verified by EEG. This is despite the fact that performing an EEG in such cases is considered to be the gold standard [24] and is recommended by national Swedish guidelines on the clinical management and diagnosis of such seizures [20]. However, the majority of the infants with neonatal seizures had received an aEEG or conventional EEG). Most neonatal seizures occur during the first three days of life [3, 6, 8], and it is conceivable that seizures associated with modifiable obstetric factors would be present in the first 24–48 hours after birth. Unfortunately, we had no information on the timing of seizures or details about their characteristics or preceding events.

Another limitation was the subjective assessment of Apgar score, comprising interobserver variability [25, 26]. However, agreement is strong for all components in term infants, except color [26], and the internal validity is considered good. We had no blood gas data at birth, which means that there may be cases of mild transient birth asphyxia in our cohort. In addition, we did not have information about indications for cesarean section or operative vaginal deliveries. Further, we acknowledge the possibility of additional confounding factors that could not be considered due to the limited number of variables in the registers.

### Interpretation

We identified several risk factors for neonatal seizures in vigorous term-born infants and, to a large extent, these corresponded with those found in infants who develop hypoxic ischemic encephalopathy [27]. Therefore, the preventive measures that have been discussed with regard to hypoxic ischemic encephalopathy may also be relevant for the population covered by our study [28–30]. Associations between intrapartum asphyxia and neonatal seizures have been reported [10, 12, 31]. However, most studies of term-born neonates with seizures and concomitant perinatal asphyxia have not presented separate findings for those without asphyxia. Our unique cohort of vigorous infants with seizures but no diagnosis of significant birth asphyxia, showed a three-fold increased risk for seizures when there had been intrapartum fetal distress. Previous studies have found associations between obstetric emergencies and asphyxia, hypoxic ischemic encephalopathy, and adverse infant outcomes [27, 32, 33]. To the best of our knowledge, this is the first study to report that obstetric emergencies, in particular shoulder dystocia and placental abruption, also predicted neonatal seizures also in vigorous infants.

Only a few previous studies have assessed maternal height as a risk for neonatal seizures [13, 32]. In our study, both short maternal stature and obesity were associated with seizures, with a dose-response pattern. The risk increased with both decreasing maternal height and increasing BMI. A high BMI has previously been shown to increase the risk of hypertensive disorders, gestational diabetes, and fetal macrosomia [34]. Other studies have also reported that labor, high BMI and short maternal height were associated with a number of problems during labor, namely cephalopelvic disproportion [35], labor dystocia [36], shoulder dystocia [37], acidosis [38, 39], and the need for Cesarean sections [40, 41]. While maternal height cannot be altered, public health strategies are needed to prevent the epidemic in increased overweight and obesity in the pregnant women [42] and the adverse impact that weight issues have on maternal and infant health.

In keeping with previous studies [5, 10], diabetes mellitus was associated with seizures and diabetic infant macrosomia could be the plausible mechanism for birth-related complications and adverse outcomes [43]. This was supported by the fact that our sensitivity analysis, which only included infants born AGA, resulted in an attenuated association between seizures and maternal diabetes. This indicated that the actual risk may have been related to fetal weight *per se* and with the increased associated risk for shoulder dystocia in neonates born LGA [37]. Decreased birth weights in term neonates have also been associated with neonatal seizures [44, 45] and our study echoed these findings, as we found being born SGA was a risk factor. Diabetes with vascular disease has been associated with fetal growth restriction [46] and diabetes and hypertensive disorders during pregnancy have been associated with placenta-mediated growth restrictions [47]. Furthermore, one study reported that children had a higher incidence of decreased birthweight if their mothers had epilepsy, when compared with those who did not [48]. The sensitivity analyses in our study indicated that the risk for seizures was to some extent associated with birthweight and maternal epilepsy.

Our findings were also consistent with previous studies that showed that both emergency and prelabor Cesarean sections predicted neonatal seizures [5, 10, 31, 45]. Cesarean sections before the onset of labor have been shown to minimizes the risk for fetal asphyxia, but did not rule out other etiologies for seizures [27]. We also found that operative vaginal deliveries increased the risk for seizures, in line with other studies [5, 31, 45]. The authors of one large cohort study reported that vacuum delivery was a predictor of seizures, especially in women with short maternal stature [49]. The associations that we found between neonatal seizures and emergency Cesarean sections and operative vaginal deliveries may have been confounded by indication for these procedures, as they are often performed due to fetal distress.

## Conclusions

This study found that obstetric emergencies, intrapartum fever, chorioamnionitis and intrapartum fetal distress were important predictors for neonatal seizures in vigorous term-born infants. Other factors included hypertensive disorders, maternal weight and height and intrapartum factors that indicated a difficult labor. A clinical implication of our study is that neonatologists should consider extra surveillance of infants who are exposed to these risk factors, as they could face an increased risk for seizures, even if they are born vigorous and at term. The extent to which such measures would reduce the occurrence of neonatal seizures needs to be evaluated in future studies. Furthermore, the need for general public health measures that address overweight and obesity in women of childbearing age cannot be overstated.

## Supporting information

S1 Table Sensitivity analysis that focused on infants with birth weights that were appropriate for gestational age. Frequencies, crude and adjusted odds ratios for neonatal seizures are presented by demography and antepartum characteristics.
(DOCX)

S2 Table Sensitivity analysis that focused on infants with birth weights that were appropriate for gestational age. Frequencies, crude and adjusted odds ratios for neonatal seizures are presented by intrapartum factors.
(DOCX)
