# Supplementary material for: Risk factors for seizures in the vigorous term neonate: A population-based register study of singleton births in Sweden
Source: PLoS One. 2022 Feb 17;17(2):e0264117. doi: 10.1371/journal.pone.0264117 (PMC8853521; doi:10.1371/journal.pone.0264117)
Supplement: S1 Table — Frequencies, crude and adjusted odds ratios for neonatal seizures are presented by demography and antepartum characteristics. (DOCX) [file pone.0264117.s001.docx]

**S1 Table.** Sensitivity analysis including infants with birth weight adequate for gestational age. Frequencies, crude and adjusted odds ratios for neonatal seizures by demography and antepartum characteristics.

| **Factors** | **Total number N=620 299** | **Seizures**  **N= 475** | **Rate /1000** | **Crude OR (95% CI)** | **Adjusted OR^1^ (95% CI)** |
| --- | --- | --- | --- | --- | --- |
| Parity |  |  |  |  |  |
| Nulliparity | 270 870 | 280 | 1.0 | 2.2 (1.7-2.7) | 2.2 (1.8-2.7) |
| Parous without previous CS | 272 628 | 129 | 0.5 | 1.0 | 1.0 |
| Parous with previous CS | 60 179 | 58 | 0.9 | 2.0 (1.5-2.7) | 1.9 (1.4-2.7) |
| Missing | 16 622 | 8 |  |  |  |
| Maternal height (cm) |  |  |  |  |  |
| ≤ 155 | 20 470 | 25 | 1.2 | 1.7 (1.1-2.6) | 1.7 (1.1-2.6) |
| 156–160 | 98 612 | 95 | 0.9 | 1.4 (1.1-1.7) | 1.3 (1.0-1.7) |
| 161-172 | 379 226 | 268 | 0.7 | 1.0 | 1.0 |
| >172 | 99 826 | 63 | 0.6 | 0.9 (0.7-1.2) | 0.9 (0.7-1.2) |
| Missing | 11 165 | 24 |  |  |  |
| Early pregnancy BMI (kg/m²) |  |  |  |  |  |
| <18.5 | 14 797 | 6 | 0.4 | 0.6 (0.3-1.3) | 0.6 (0.3-1.3) |
| 18.5-24.9 | 348 858 | 240 | 0.7 | 1.0 | 1.0 |
| 25-29.9 | 150 321 | 126 | 0.8 | 1.2 (0.9-1.5) | 1.2 (0.9-1.5) |
| 30-34.9 | 50 231 | 48 | 0.9 | 1.4 (1.0-1.9) | 1.3 (0.9-1.8) |
| 35-39.9 | 15 153 | 16 | 1.1 | 1.5 (0.9-2.5) | 1.6 (0.9-2.6) |
| >40 | 5 182 | 9 | 1.7 | 2.5 (1.3-4.9) | 2.6 (1.3-5.0) |
| Missing | 35 757 | 30 |  |  |  |
| Infertility treatment | 18 248 | 23 | 1.3 | 1.6 (1.1-2.5) | 1.5 (1.0-2.3) |
| Epilepsy | 3 084 | 5 | 1.6 | 2.1 (0.9-5.1) | 2.1 (0.9-5.1) |
| Hypertensive disorders ^a^ | 22 673 | 40 | 1,7 | 2.4 (1.7-3.6) | 2.0 (1.4-2.8) |
| Diabetes mellitus^b^ | 7 751 | 9 | 1.2 | 1.5 (0.8-2.9) | 1.4 (0.7-2.7) |

^a^ Chronic hypertension, gestational hypertension, preeclampsia, or eclampsia

^b^ Pregestational or gestational diabetes mellitus

^1^Adjusted for parity, maternal height, BMI, gestational age and birth year

CS: Cesarean section; BMI: Body Mass Index
