# Supplementary material for: Risk factors for seizures in the vigorous term neonate: A population-based register study of singleton births in Sweden
Source: PLoS One. 2022 Feb 17;17(2):e0264117. doi: 10.1371/journal.pone.0264117 (PMC8853521; doi:10.1371/journal.pone.0264117)
Supplement: S2 Table — Frequencies, crude and adjusted odds ratios for neonatal seizures are presented by intrapartum factors. (DOCX) [file pone.0264117.s002.docx]

**S2 Table.** Sensitivity analysis including infants with birth weight adequate for gestational age. Frequencies, crude and adjusted odds ratios for neonatal seizures by intrapartum factors.

|  | **Total number N=620 299** | **Seizures N=475 Rate/ 1000** | | **Crude OR (95%CI)** | **Adjusted OR^1^ (95%CI)** |
| --- | --- | --- | --- | --- | --- |
| Induced labor | 85 900 | 109 (22.9) | 1.3 | 1.9 (1.5-2.3) | 1.7 (1.4-2.2) |
| Gestational age, weeks |  |  |  |  |  |
| 37-40 | 454 419 | 330 (69.5) | 0.7 | 1.0 | 1.0 |
| ≥ 41 | 120 875 | 95 (20.0) | 0.8 | 1.1 (0.9-1.4) | 1.0 (0.8-1.3) |
| ≥ 42 | 45 005 | 50 (10.5) | 1.1 | 1.5 (1.1-2.1) | 1.3 (1.0-1.8) |
| Dystocia | 71 925 | 96 (20.2) | 1.3 | 1.9 (1.5-2.4) | 1.5 (1.2-1.9) |
| Meconium | 5 471 | 16 (3.4) | 2.9 | 3.9 (2.4-6.5) | 3.8 (2.3-6.2) |
| Intrapartum fever and/or chorioamnionitis | 5 545 | 19 (4.0) | *3.4* | 4.6 (2.9-7.3) | 3.5 (2.2-5.6) |
| Position at birth |  |  |  |  |  |
| Occiput anterior | 556 215 | 399 (85.8) | 0.7 | 1.0 | 1.0 |
| Occiput posterior | 27 269 | 36 (7.7) | 1.3 | 1.8 (1.3-2.6) | 1.8 (1.9-2.4) |
| Breech lie | 14 445 | 13 (2.8) | 0.9 | 1.3 (0.7-2.2) | 1.2 (0.6-2.0) |
| Other | 10 327 | 17 (3.7) | 1.6 | 2.3 (1.4-3.7) | 1.8 (1.1-2.0) |
| Missing | 12 043 | 10 (2.1) |  | *-* | - |
| Mode of delivery |  |  |  |  |  |
| Spontaneous vaginal | 480 791 | 280 (60.3) | 0.6 | 1.0 | 1.0 |
| Vacuum or forceps | 43 785 | 70 (15.1) | 1.6 | 2.7 (2.1-3.6) | 2.1 (1.6-2.8) |
| Emergency CS | 39 924 | 68 (14.7) | 1.7 | 2.9 (2.2-3.8) | 2.3 (1.7-3.1) |
| Prelabor CS | 45 609 | 46 (9.9) | 1.0 | 1.7 (1.3-2.4) | 1.5 (1.0-2.1) |
| Missing | 10 190 | 11 (2.3) |  |  |  |
| Fetal distress | 41 653 | 93 190.6) | 2.2 | 3.4 (2.7-4.3) | 2.9 (2.3-3.7) |
| Obstetric emergency | 2 817 | 12 (2.5) | 4.3 | 5.7 (3.2-10.1) | 4.6 (2.4-8.6) |
| Shoulder dystocia | 877 | 6 (1.3) | 6.8 | 9.9 (4.1-20.4) | 7.7 (3.2-18.6) |
| Placental abruption | 852 | 4 (0.8) | 4.7 | 6.2 (2.3-16.6) | 4.5 (1.4-14.1) |
| Head circumference > 37 cm | 89 087 | 95 (21.0) | 1.1 | 1.6 (1.3-1.9) | 1.4 (1.1-1.8) |
| Male sex | 316 637 | 268 (56.4) | 0.8 | 1.2 (1.0-1.5) | 1.2 (1.0-1.5) |

Premature rupture of membranes, cord complication and birthweight not shown in table.

Values are presented as n (%) or mean ± SD

^1^ Adjusted for parity, maternal height, BMI, gestational age and birth year

CS: Cesarean section
